# Design of Polymeric Films for Antioxidant Active Food Packaging

**DOI:** 10.3390/ijms23010012

**Published:** 2021-12-21

**Authors:** Wing-Fu Lai

**Affiliations:** 1 Department of Applied Biology and Chemical Technology, Hong Kong Polytechnic University, Hong Kong, China; rori0610@graduate.hku.hk; 2 Ciechanover Institute of Precision and Regenerative Medicine, The Chinese University of Hong Kong (Shenzhen), Shenzhen 518172, China

**Keywords:** food packaging, polymers, films, food preservation, antioxidants, quality control, oxidation

## Abstract

Antioxidant active food packaging can extend the shelf life of foods by retarding the rate of oxidation reactions of food components. Although significant advances in the design and development of polymeric packaging films loaded with antioxidants have been achieved over the last several decades, few of these films have successfully been translated from the laboratory to commercial applications. This article presents a snapshot of the latest advances in the design and applications of polymeric films for antioxidant active food packaging. It is hoped that this article will offer insights into the optimisation of the performance of polymeric films for food packaging purposes and will facilitate the translation of those polymeric films from the laboratory to commercial applications in the food industry.

## 1. Introduction

Food quality is determined by consumer acceptability, which is largely affected by the taste, appearance, and aroma of a food product. Quality may deteriorate when different food components undergo oxidation [1]. One of these components is proteins [2,3]. Oxidation of proteins can decrease the juiciness and increase the toughness of meat products [4,5]. The off-flavour of irradiated meat is also thought to be the result of oxidation reactions upon irradiation of sulphur-containing amino acids [6]. Lipids are another food component that is susceptible to oxidation [7,8,9,10,11]. Lipid oxidation is one of the major causes of food deterioration [3]. It is especially serious for food products with a high lipid content [7,12]. Examples of these products include nuts, fish oils, meat, and fishery products. Lipid oxidation severely affects the sensory attributes of the products, leading to rancidity, formation of toxic aldehydes, and loss of nutritional value [7,13]. The latter is especially important for functional foods with a high content of polyunsaturated fatty acids (PUFAs). This is because PUFAs have been linked to cardiovascular protection. Degradation of these fatty acids during lipid oxidation can lead to a decline in the health-promoting effect [7,12]. Chemically, lipid oxidation is initiated when an alkyl radical is formed upon removal of a hydrogen atom from an unsaturated fatty acid [14]. Because the reaction for the generation of an alkyl radical is not thermodynamically favourable, it is often initiated in the presence of singlet oxygen (which can be generated in response to the presence of transition metals, exposure of the food product to UV, or temperature change), or pigments that can serve as photosensitisers [15]. Furthermore, the alkyl radical can react with O_2_ to generate a peroxyl radical that subsequently abstracts a hydrogen atom from another unsaturated fatty acid, leading to the formation of a new alkyl radical as well as a lipid hydroperoxide [16]. Lipid hydroperoxide is a major primary product of lipid oxidation. Although it is odourless and tasteless, it can decompose to compounds that give off-flavours in the presence of heat, light, or metal ions.

To maintain product quality, various strategies for reducing food oxidation have been adopted [12,17,18]. One strategy is to add antioxidants directly to food products [18]. While this method is easy to apply, once the antioxidants are consumed in the oxidation reaction, the extent of protection obtained by the food is reduced, causing faster degradation of food quality. Another strategy is to combine high-barrier packaging materials with vacuum or modified-atmosphere food packaging [19,20,21]. This method can reduce the oxygen content inside the package even though achieving total elimination of oxygen is technically challenging [22]. Recently, the concept of active food packaging has been developed [23,24,25,26], with various food companies and research institutions contributing research efforts to the development of active packaging films [27,28,29] and some packaging technologies reaching commercial use [30]. Active food packaging can be classified into two types. The first involves the incorporation of independent devices (such as pads or sachets in which chemicals or mixtures that can release or retain a specific gas or vapour so as to promote the preservation or quality of a food product) into the conventional passive food package. In addressing oxidation, oxygen scavengers are often involved. Common examples include iron or ferrous oxide fine powders although other alternatives (such as sulphites, catechol, ascorbic acid, and glucose oxidase) have sometimes been adopted [31]. Another type of active food packaging involves the use of food packaging materials into which an active agent is incorporated. Because many oxygen scavengers are not edible and may even be toxic, compared to the first approach in which independent devices are adopted, and hence may cause safety concerns if the pads or sachets containing the chemicals are broken, the use of safe and active packaging materials may prevent this problem from occurring. In addition, the combination of both the packaging function and active function into a single film can reduce the number of steps in the food manufacturing process and enhance the efficiency of food production. With regard to the practical potential of active food packaging in food preservation, the objective of this article is to present the latest advances in the design of polymeric packaging films with antioxidant properties for combating oxidation in food products. Directions and challenges for the commercial translation of films for antioxidant active food packaging in future research will also be discussed.

## 2. Strategies of Antioxidant Incorporation into Polymeric Films

The incorporation of antioxidants into polymeric packaging films can be achieved either physically or chemically. While each of these methods has its advantages and disadvantages (Table 1), the generated films have also been successfully demonstrated for possible use in antioxidant active food packaging to different extents.

### 2.1. Physical Means

**Figure 1:**
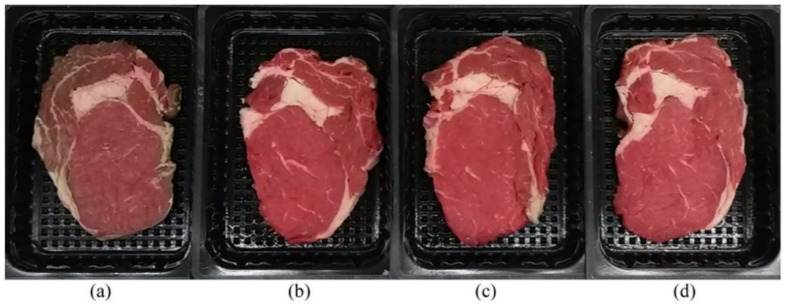
Images for fresh beef packaged with different films under modified atmosphere for 14 days at 2 ± 1 °C: (**a**) an uncoated OPP film, (**b**) an AC/S-coated OPP film with the content of EU being 0.32 ± 0.03 g m^−2^, (**c**) an AC/S-coated OPP film with the content of EU being 0.40 ± 0.14 g m^−2^, and (**d**) a CA-coated OPP film with the content of EU being 0.65 ± 0.08 g m^−2^. Reproduced from [33] with permission from Elsevier B.V.

For the loading of antioxidants into polymeric films, most of the reported studies mix the antioxidant with the film-forming solution prior to film generation. This method is compatible with all major methods of film production, ranging from solution casting to melt extrusion. In addition, because agent loading and film formation can be integrated into a single step, the film generation process is simplified. Nevertheless, because active ingredients are present inside the film, their antioxidant activity may be impeded if they are not freely accessible to participate in fighting against lipid radicals when the food product is under oxidative stress. To tackle this problem, recently, some efforts have been made to generate antioxidant films by coating an active layer onto the surface of a film substrate. The technical feasibility of this method has been demonstrated by Lee et al. [32] who dipped a poly(lactic acid) (PLA)-based film into a solution containing the active ingredient (e.g., morin, quercetin, caffeic acid, and curcumin) to generate a film with antioxidant properties. Although the reported film has only been exploited for biomedical applications, such a method may also be applicable for the generation of films for food packaging. A similar approach has been used by Navikaite-Snipaitiene et al. [33], who have surface-coated a corona-treated oriented polypropylene film (OPP) with either cellulose acetate (CA) or acrylic component/hydrophobically modified starch (AC/S) by using an automatic film applicator. Clove essential oil (CL) or eugenol (EU) was used as the antioxidant agent. The coated films have been demonstrated to effectively retard the rate of lipid oxidation and enhance the red colour stability of beef steaks during storage (Figure 1).

**Figure 2:**
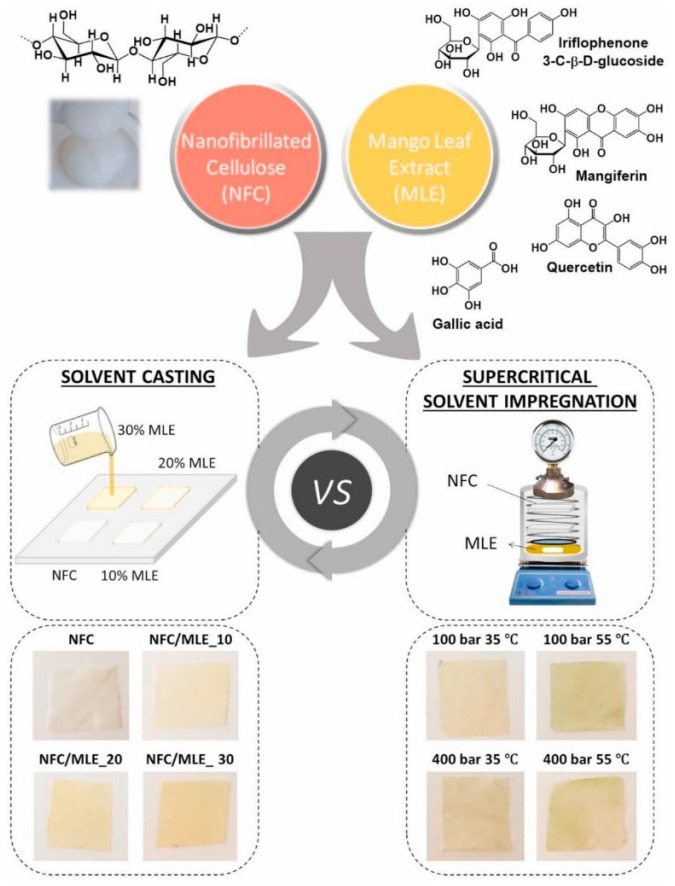
A schematic diagram illustrating the preparation of NFC/MLE-based films via either solvent casting or SSI. Reproduced from [35] with permission from Elsevier B.V.

To further enhance the coating efficiency, supercritical solvent impregnation (SSI) has recently been adopted to generate polymeric films coated with antioxidants. Using the SSI process, active agents can be easily impregnated into the polymeric matrix of a packaging film under mild conditions by using supercritical carbon dioxide as the solvent. SSI has been shown to eliminate some drawbacks (including the uneven distribution of active ingredients in the fabricated film, and the evaporation of active ingredients during film production) associated with solution casting [34]. The success of this approach in the fabrication of antioxidant packaging films has been demonstrated by Bastante et al. [35] who produced films composed of nanofibrillated cellulose (NFC) and mango leaf extract (MLE) via both SSI and solvent casting (Figure 2). The MLE-containing films generated by CO_2_-assisted impregnation have been found to be thermally stable up to 250 °C. In addition, they exhibited good mechanical strength and UV-blocking capacity. Importantly, unlike solution casting which causes the MLE components to be trapped in the bulk of the polymeric matrix of the film, SSI enables the MLE components to be adsorbed mostly at the film surface [35]. This enables a higher rate of migration of the components from the film to the food, and hence higher antioxidant capacity [35].

Despite the encouraging potential mentioned above, proper adherence of the active coating to the film substrate is required if the antioxidant is supposed to be on the surface of the film. This is largely determined by the affinity of the film substrate for the coating agent and can be improved by treating the film substrate chemically (e.g., the use of primers) or physically (e.g., UV irradiation, and corona or flame discharge) immediately before the coating process. Furthermore, the antioxidant in the film generated by this method is present mainly on the film surface without being protected by the polymeric matrix, and it is expected to be released more rapidly. Once the antioxidant is fully released, the film may lose its antioxidant capacity. Therefore, the sustainability of the antioxidant capacity of the film must be further evaluated to determine the period of effective protection conferred by the film. Finally, the extent of migration from the packaging film to food production is more significant if the antioxidant is on the film surface. If the antioxidant is not a “generally regarded as safe” (GRAS) substance, safety concern may be raised if the antioxidant can migrate freely into the food product.

### 2.2. Chemical Means

Chemical antioxidant incorporation involves the covalent linkage of functional compounds with antioxidant properties into the film-forming agent. The use of this method has been demonstrated by Fazio et al. [36], who first performed enzymatic transesterification to enable the primary hydroxyl group of tyrosol or hydroxytyrosol being regioselectively acylated by acrylic acid methyl ester under the action of *Candida antarctica* lipase B, followed by polymerisation of the products (viz., tyrosyl acrylate and hydroxytyrosyl acrylate) to generate poly(tyrosyl acrylate) (PTA) and poly(hydroxytyrosyl acrylate) (PHTA). Finally, the technique of spin coating was adopted to generate thin films by using acetone solutions of PTA and PHTA. The films were found to inhibit ascorbic acid oxidation in fresh orange juice.

While physical methods are commonly used to load antioxidants into packaging materials, they often lead to excessive loss of antioxidants during application and weaken the physicochemical properties of the packaging material. To avoid these problems, antioxidants are sometimes immobilised onto the surface of a packaging material through covalent immobilisation, which on one hand prevents the migration of the antioxidants from the packaging film to the foods and, on the other hand, have little influence on the thermomechanical properties of the bulk film [37]. To enable covalent immobilisation, the surface of the film was pretreated to introduce active groups. Common methods of film pretreatment include UV irradiation, ionised gas treatment, and wet chemical treatment [38]. Among these three methods, wet chemical treatment is less commonly adopted because it involves the use of concentrated corrosive agents which can be hazardous when handling. In contrast, UV irradiation and ionised gas treatment are more environmentally friendly. Importantly, the former can be performed at a low cost and can induce crosslinking of the polymer network of the film, making the microstructure of the film more compact, thereby enabling the release of the loaded antioxidants in a more controlled manner [39].

In addition to modifying the film surface using plasma treatment to enhance antioxidant incorporation into the film via chemical means [40], the structure of the antioxidant can be modified with desired functional groups to render the agent capable of either grafting onto the polymer molecules or directly polymerising with comonomers or crosslinkers. Compared to having the modified agent to polymerise directly with comonomers, introduction of a crosslinker on one hand increases the type of functional groups usable for the design and production of the packaging film, and on the other hand reduces steric resistance during film fabrication [16]. In an earlier study, the H_2_O_2_/ascorbic acid redox initiator system was adopted for the free-radical-induced covalent grafting of phenolic compounds with pectin to generate bioactive polymer films [41]. Compared with those generated by crosslinking of phenolic compounds and pectin using calcium ions, the films generated using free radical covalent grafting have been shown to have 4-fold higher activity of 2,2-diphenyl-1-picrylhydrazyl (DPPH) radical inhibition [41].

## 3. Selection of Antioxidant Agents to be Incorporated into Polymeric Films

Over the years, different agents have been exploited for use in the development of antioxidant packaging films. These agents can be classified based on their origin (natural or artificial) or composition (i.e., being a single purified compound or being a mixture of chemicals). By integrating these two classification systems, the antioxidant agents used in antioxidant active food packaging are grouped into three categories: plant extracts, purified compounds from natural sources, and synthetic agents.

### 3.1. Plant Extracts

Plant extracts are a major class of additives used for the fabrication of antioxidant food packaging films. One example is MLE, which is rich in mangiferin, gallic acid, glucosides, and other phenolic compounds and hence exhibits excellent antioxidant capacity [42]. It was previously adopted by Belizón et al. [43], who applied SSI to impregnate the MLE into a solid hydrophobic and non-biodegradable multilayer film consisting of poly(ethylene terephthalate) (PET) and polypropylene (PP) for the preservation of perishable foods such as fruits and vegetables. Furthermore, upon incorporation into soy protein isolate and fish gelatin, food packaging films exhibiting high free radical scavenging capacity have been generated [44]. Recently, MLE has been incorporated into chitosan films, with glycerol being adopted as a plasticiser. The antioxidant activity of the films has been reported to be positively related to the concentration of MLE present. The films have been exploited for cashew nut preservation and have effectively inhibited fatty acid oxidation [45]. In addition to MLE, the extract of pine needles (*Cedrus deodara*) has served as a source of polyphenolic compounds and has been incorporated into chitosan films to combat the oxidation of food components [46]. These results demonstrate the effectiveness of the extracts rich in phenolic compounds in serving as an active ingredient in antioxidant food packaging.

Since the turn of the last century, the leaf extract of *Moringa oleifera* L. (also known as benzolive tree, horseradish tree, drumstick tree, and ben oil tree) has attracted attention in research on antioxidant food packaging. *M. oleifera* L. is a drought-resistant tree with a high phenolic and flavonoid content [47]. It belongs to the Moringaceae family and is native to Afghanistan, Bangladesh, Pakistan, and India [48]. The use of this tree in the development of antioxidant packaging films was exemplified by an earlier study, which incorporated *M. oleifera* leaf extract into a fish skin gelatin film to generate an antioxidant film for Gouda cheese preservation [49]. *M. oleifera* leaf extract, combined with chitosan and carboxymethyl cellulose (CMC), has also been used to fabricate edible coatings to enhance fruit quality in avocado (*Persea americana* Mill) [50]. The coating has not only been shown to reduce fruit weight loss and to slow down the respiration process, but it can also increase the fruit phytochemical characteristics, decrease the activity of polyphenol oxidase (PPO), and reduce the extent of lipid peroxidation [50]. More recently, Ju et al. have also incorporated leaf extract into khorasan wheat starch (KWS) films for antioxidant food packaging [51].

### 3.2. Purified Compounds from Natural Sources

**Figure 3:**
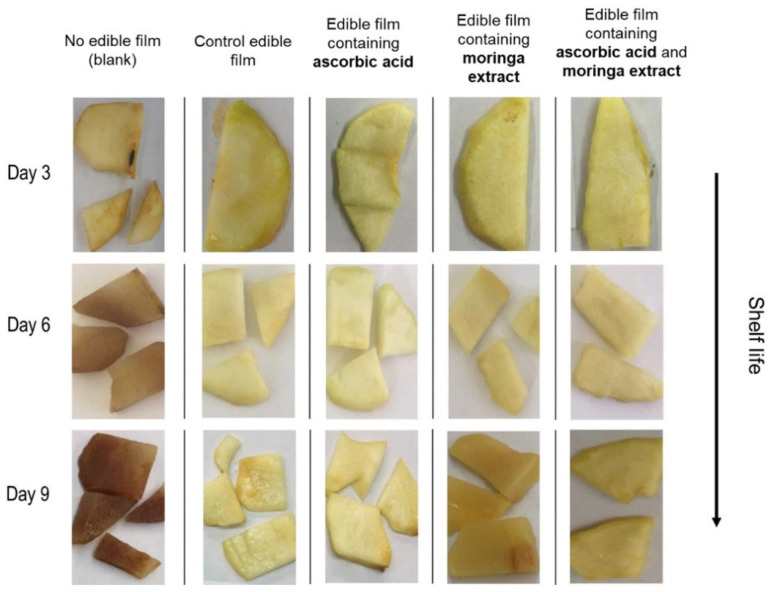
Changes in the colour of pear samples packed in different antioxidant papaya edible films. Reproduced from [53] with permission from Elsevier B.V.

Apart from plant extracts, purified small molecular compounds found in fruits and vegetables have been adopted for the development of antioxidant packaging films. One example is ascorbic acid, which is a natural compound found in vegetables and citrus fruits. It is widely used to process fresh fruits to prevent enzymatic browning [52]. The possible use of this agent in the generation of antioxidant packaging films has been partially demonstrated by Rodríguez et al. [53], who generated edible films from the purée of papaya (*Carica papaya* L.) to inhibit the browning of minimally processed pear (*Pyrus communis* L.) [53]. The films incorporated with ascorbic acid, with or without the leaf extract of *M. oleifera* L., successfully retarded the browning of pear samples (Figure 3) [53]. Another example is curcumin, which has been shown to exhibit not only antimicrobial and antioxidant properties [54,55,56,57,58] but also the capacity to change colour in response to changes in pH [59,60]. Over the years, numerous antioxidant packaging films have been generated from the use of curcumin, with macromolecules such as k-carrageenan [61], gelatin [62], corn zein [63], chitosan [64], and tara gum [60] being adopted as the host matrices.

While most of the active ingredients used in film development for antioxidant food packaging come from botanical sources, antioxidants obtained from animal sources have also been adopted. A good example is melanin, a ubiquitous biological pigment found widely in the eyes, hair, skin, and brain of living animals [65,66]. Natural melanin contains eumelanin and pheomelanin. Both are obtained from the precursor dopaquinone, which is generated via the enzymatic oxidation of tyrosine. Recently, melanin nanoparticles have been used in film making for the generation of an antioxidant food packaging film [67]. During synthesis, melanin precipitate was first obtained from the squid ink paste, followed by drying in an oven at 50 °C to obtain melanin powder. The powder was then dissolved in an NaOH solution. Alkali-soluble melanin was collected by centrifugation, and added dropwise into 1 M HCl to obtain melanin nanoparticles. The nanoparticles are spherical in shape and have an average diameter of approximately 100 nm [67]. They were dispersed in distilled water and mixed with a gelatin solution to form a nanocomposite film, whose antioxidant activity has been shown to be positively related to the concentration of the nanoparticles present [67].

### 3.3. Synthetic Agents

Inorganic agents have been used as antioxidants, too. ZnO nanoparticles are a good example of such antioxidants and have been adopted in food packaging as antimicrobial agents and as UV light absorbers [68,69]. The amount of ZnO nanoparticles permitted to be added to the polymer matrix of food packaging films is approximately 2–10% [68]. In the development of antioxidant food packaging films, the hydroxyl groups on the nanoparticle surface are regarded as an important source of the antioxidant activity exhibited by the nanoparticles [70]. In the DPPH assay, which is a commonly used assay in the literature to evaluate the antioxidant properties of an active packaging film, the transfer of electron density at the oxygen atom in ZnO nanoparticles to the odd electron at the nitrogen atom in DPPH leads to a decrease in the n→π* transition intensity and hence in the absorbance at 517 nm [70]. This confirms the antioxidant capacity of the packaging films.

Food additives approved by the Food and Drug Administration (FDA) in the US for use as antioxidants have also been incorporated into the film matrix for antioxidant active food packaging. Representative examples include butylated hydroxyanisole (BHA), butylated hydroxytoluene (BHT), and tertiary butylated hydroquinone (TBHQ). These additives have been incorporated into polypropylene-based films and have been exploited for possible use in food protection [71]. An increase in the concentration of BHA, BHT, and TBHQ led to an increase in the scavenging activity of the film, with the antioxidant capacity of BHT and TBHQ found to be higher than that of BHA in films containing 3% of the antioxidant [71]. It is important to note that although synthetic antioxidants have been adopted in the development of antioxidant packaging films, the possible toxicity caused by the migration of these agents from the films to the food products raises safety concern. This problem is compounded by the fact that the extent of migration is determined by diverse factors [72], including the physical properties of the additive, the concentration of the additive in the film, nature of the food, temperature, time of contact, and properties of the packaging material. Therefore, extensive evaluation of the extent of migration must be performed on a case-by-case basis. Strict statutory control must also be imposed before the film can be applied in practical use.

## 4. Translation from the Laboratory to Food Industry

Multiple steps are involved from the time a film is first designed to the time when the film is manufactured and used by end-users. Failure can occur at one of these many steps, which can compromise technology transfer [73,74]. For instance, even if an innovative film is designed and tested in a laboratory to be effective in protecting the food product from oxidation, if the method of film production cannot be easily scaled up or if the quality of the antioxidant cannot be guaranteed throughout the year, the film may not be ideal for further commercialisation [73,75]. This is particularly true because most of the efforts devoted to the development of antioxidant polymeric films are performed in academic settings. Many of the studies are indeed proof-of-concept in nature, making the industry difficult to adopt the reported works for commercialisation. Furthermore, academic research has largely emphasised novelty. Although films with new compositions and structures may be attractive for academic publication, they might not be compatible with existing industrial processes or systems. This will put industrial producers at a high risk when they invest in new technologies or processes to work on a laboratory-tested idea. To address the gap between knowledge acquisition and industrial use, academic researchers may consider the industrial needs as well as the cost and technical feasibility during the manufacturing and marketing of innovative design. This can be partially achieved by strategic partnerships between knowledge-driven researchers in the academic setting and market-driven industrial partners [73].

In addition, when the film is used in the food industry, it is expected to experience potentially remarkable variations in temperature and humidity during food processing and storage, or even tear and wear during rough handling. The extent of such variations is sometimes much larger than what the laboratory can imitate or predict. This is compounded by the fact that most of the studies on antioxidant active food packaging have only tested the antioxidant capacity of the film using chemical tests such as the DPPH assay and the 2,2′-azino-bis(3-ethylbenzothiazoline-6-sulfonic acid) radical scavenging assay. Even in studies that adopt food models to evaluate the performance of the film, the variety of food models used is limited. This hinders the technology transfer and industrial application of the reported film because the composition of the food, in fact, affects the rate of release of the antioxidant from the film, leading to variations in the efficiency of food protection based on antioxidant food packaging. This has been confirmed by a recent study [76], which examined the influence of both water activity and viscosity of the food simulant on the antioxidant activity and release kinetics of phenolic acids embedded in gelatin/chitosan packaging films. The diffusion and convection coefficients of phenolic acids have been found to correlate with the viscosity of the release medium [76], whose water activity and solute nature have also been shown to lead to changes in the partition coefficient of phenolic acids [76]. The incomplete understanding of the strength and limitations of a film is one reason why a film that performs well in a laboratory sometimes does not succeed in turning into a product.

## 5. Perspectives and Concluding Remarks

Antioxidant active food packaging can prolong the shelf life of foods by retarding the rate of oxidation reactions experienced by food components. As discussed in the sections above, significant advances in the design and development of packaging films loaded with a large diversity of antioxidants have been achieved over the last several decades, yet few challenges remain to be solved before antioxidant active food packaging films can be effectively translated from the laboratory into commercial applications. One challenge is the poor understanding of the interactions between antioxidants and other film components during film fabrication. The bulk properties of the film may change when antioxidants are added to the film. If the generation of the film involves the use of other additives such as plasticisers and colouring agents, the release kinetics of these additives may be changed, leading to the migration of these agents to food products and causing safety concern. For this, more studies are required to decipher and predict the interactions among different additives in a film so that films with better properties can be designed.

The development of effective antioxidant active food packaging films is compounded by the difficulty of determining and optimising the amount and concentration distribution of the antioxidant in the packaging film. Uneven distribution or a suboptimal concentration of the antioxidant can hamper the efficiency of the film in food protection. Furthermore, the polymeric matrix of the film needs to be properly designed so that the release kinetics of the antioxidant match the kinetics of oxidation of food components. Over the years, various studies have been performed to apply mathematical models of mass transfer to examine the release kinetics of bioactive agents from the polymeric matrix; however, most of these studies have been performed by placing the matrix in a liquid environment [77,78]. The release kinetics observed in a liquid environment may be different from that when a polymeric film is applied to package solid foods. Despite the challenges highlighted above, as presented in the literature, recent advances in the development of antioxidant active food packaging films have successfully enabled shelf-life extension and food quality maintenance. With the continuous progress in technologies for film fabrication and engineering, as well as the emergence of innovative materials for food packaging, it is expected that consumer acceptance of antioxidant active food packaging will continue to grow, and the role played by related packaging films will become increasingly significant in the food industry in the future.

## Figures and Tables

**Table 1:** Pros and cons of physical and chemical means of antioxidant incorporation.

	Working Principle	Pros	Cons
Physical means	The antioxidant either is entrapped physically by the polymeric matrix or adheres directly to the film	‑ Easy to operate ‑ Have an extensive track record of use in the literature for film design ‑ Ideal to applications in which the protective effect of the film relies on the release of the incorporated antioxidant	‑ Safety concern due to the possible migration of the incorporated antioxidant (particularly those whose level in foods is to be tightly regulated) from the packaging material to the food ‑ Effective antioxidant incorporation is limited to the antioxidant which is physically compatible with the film ‑ Lower stability of the antioxidant-incorporated film for storage
Chemical means	Covalent bonds are adopted to link the antioxidant either to a pre-generated polymeric film or to the monomer before polymerisation	‑ Effectively reduce the migration of the incorporated antioxidant from the packaging material to the food product ‑ The efficiency of antioxidant incorporation is not influenced by the physical compatibility of the antioxidant with the film ‑ Higher stability of the antioxidant-incorporated film for storage	‑ Knowledge of synthetic chemistry is required for the design of the material ‑ Potentially labour-intensive and time consuming ‑ Organic solvents may be adopted during reactions and the chemical residues in the product may cause safety concern ‑ Not suitable to be used in situations where the protective effect of the film relies on the release of the incorporated antioxidant
